# Nanoparticles in gynecologic cancers: a bibliometric and visualization analysis

**DOI:** 10.3389/fonc.2024.1465987

**Published:** 2025-01-08

**Authors:** Yunzhe Zhou, Lizhang Chen, Tingting Wang

**Affiliations:** Department of Epidemiology and Health Statistics, Xiangya School of Public Health, Central South University, Changsha, China

**Keywords:** gynecologic cancers, nanoparticles, nanomaterials, bibliometric analysis, visualization analysis

## Abstract

**Background:**

Gynecological cancers are characterized by uncontrolled cell proliferation within the female reproductive organs. These cancers pose a significant threat to women’s health, impacting life expectancy, quality of life, and fertility. Nanoparticles, with their small size, large surface area, and high permeability, have become a key focus in targeted cancer therapy. The aim of this study is to review recent advancements in nanoparticles applied to gynecologic cancers, providing valuable insights for future research.

**Methods:**

We retrieved all literature on nanoparticles in gynecologic cancers from the Web of Science Core Collection (WOSCC) database between January 1, 2004, and June 4, 2024. Data analysis and visualization were conducted using R software (version 4.4.0), VOSviewer (version 1.6.19.0), and CiteSpace (version 6.1).

**Results:**

A total of 2,843 publications from January 1, 2004, to June 4, 2024 were searched. Over the past 20 years, there has been a significant increase in publications. The leading countries and institutions in terms of productivity are China and the Chinese Academy of Sciences. The most prolific author and the most co-cited author are Sood, A K and Siegel, Rl. The top journals are the *International Journal of Nanomedicine* (n=97), followed by *ACS Applied Materials & Interfaces* (n=72) and *Journal of Materials Chemistry B* (n=53). Keyword analysis shows current research focuses on two main areas: the application of nanoparticles for drug delivery and their broader applications in gynecologic cancers. Future research will likely focus on “silver nanoparticles,” “gold nanoparticles,” and “green synthesis.”

**Conclusions:**

Over the past two decades, nanoparticles have rapidly advanced in the field of gynecologic cancers. Research has primarily focused on the applications of nanoparticles in drug delivery and applications. Future trends point toward optimizing synthesis techniques and advancing preclinical studies to clinical applications, particularly for silver and gold nanoparticles. These findings provide valuable scientific insights for researchers.

## 1 Introduction

Gynecological cancers, characterized by uncontrolled cell growth in female reproductive organs, include five main types: cervical, ovarian, uterine, vaginal, and vulvar cancers (1). These malignancies pose significant threats to women’s health, impacting life expectancy, quality of life, and fertility (2). In 2024, statistics reported over 116,000 new cases of gynecological cancers (3). As one of the major cancers affecting women globally (4), gynecological malignancies not only have high incidence rates but also demand urgent improvement in prognosis (5–7). Current treatment modalities include surgery, chemotherapy, radiotherapy, and immunotherapy. Surgery is mainly applicable to early-stage solid tumors but may face risks of incomplete resection and potential tumor metastasis or implantation (8, 9). Chemotherapy is associated with cytotoxicity and low bioavailability, limiting its widespread application (10, 11). Immunotherapy has limited clinical applicability and is not applicable to tumor types with “immune suppression” or “immune exclusion.” (12).

In recent years, nanoparticles have shown potential to overcome limitations of traditional therapies and have rapidly progressed in biomedicine (13). Their advantages lie in their small size, large surface area, high permeability, and ability to effectively combine with various biomaterials. These characteristics give nanoparticles significant advantages in drug delivery and controlled release, making them increasingly favored in cancer treatment (14, 15). In the treatment of ovarian cancer, combining paclitaxel with other drugs in nanocarrier systems enables precise targeted delivery, reduces off-target toxicity, and effectively improves solubility issues (16). Furthermore, nanoparticles not only serve as drug carriers but also hold potential for direct anticancer applications. For instance, silica-coated gold (Au@SiO2) nanoparticles show promise in treating cervical cancer (17). Research has demonstrated that graphene oxide nanoparticles encapsulating chlorambucil can lower cellular toxicity and exhibit high drug loading efficiency and controlled release capabilities in treating cervical adenocarcinoma (18). Therefore, the application of nanoparticles in treating gynecological malignancies represents a frontier therapy with immense potential.

Bibliometrics is an academic discipline that employs statistical methods to provide an objective framework for tracking the evolution and structural composition of specific research fields (19, 20). It has been widely used to explore trends and hot topics across various publishing domains, including psychiatry, obstetrics, and gynecology (21, 22). To our knowledge, despite significant growth in publications related to nanoparticles in gynecologic cancers, there has been no bibliometric analysis conducted in this area. A bibliometric analysis of the application of nanoparticles in gynecologic malignancies can provide deeper insights into their role in the treatment of these cancers. This study undertook a bibliometric review and summary of literature from 2004 to 2024 pertaining to nanoparticles in gynecologic cancers. By exploring dimensions such as countries, institutions, journals, authors, and keywords associated with nanoparticles in gynecologic cancers, this analysis aims to offer valuable insights into potential gaps in the literature and guides future research directions, thereby contributing significantly to advancing the field.

## 2 Methods

### 2.1 Data source

**Figure 1:**
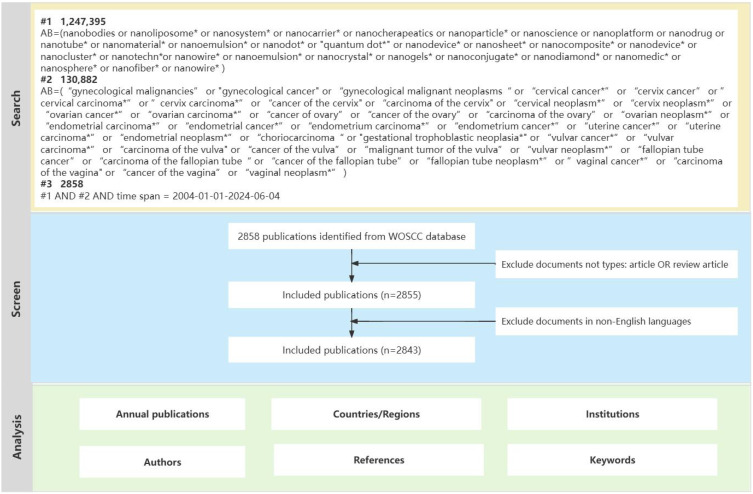
Flowchart of the publications selection.

On June 4, 2024, we conducted a comprehensive search in the Web of Science Core Collection (WOSCC) database. WOSCC, recognized as a premier academic information database, is distinguished by its selection of high-impact journals, which makes it particularly suitable for bibliometric analysis compared to other databases (23). In this study, we included only English-language publications categorized as “article” or “review”. Given the limited number of publications in the relevant field prior to 2004, the time span was set from January 1, 2004, to June 4, 2024. The specific literature selection process is depicted in **Figure 1**.

### 2.2 Data analysis and visualization

This study primarily utilized Microsoft Excel 2021, R software (version 4.4.0), VOSviewer (version 1.6.19.0), and CiteSpace (version 6.1) for visual analysis. The bibliometrix package in R software is specifically designed for bibliometric analysis (24). In this research, we utilized this package to create graphical representations of keyword counts, showcasing visual maps of hotspot development trends. VOSviewer utilizes probabilistic data standardization methods for visualizing data and constructing various network connections (25, 26). In this study, we used VOSviewer to analyze associations among countries, institutions, authors, references, and co-occurrence of keywords. CiteSpace, a robust exploration tool widely used in data visualization, employs standardized data aggregation and burst detection methods to track emerging research trends and future directions (27). In this manuscript, we employed CiteSpace to analyze centrality data of countries and institutions, overlapped journal co-citation networks, and conducted burst detection analysis of references and keywords, generating visualizations accordingly.

### 2.3 Research ethics

The data utilized in our study were obtained from publicly accessible databases containing published articles. As the study did not involve animal or human subjects, ethical approval from a committee was deemed unnecessary.

## 3 Results

### 3.1 Analysis of publications

**Figure 2:**
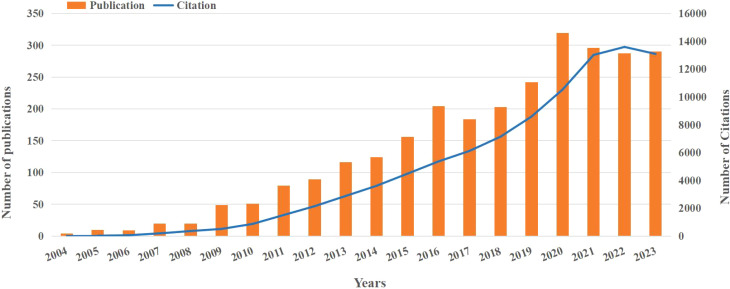
The number of publications and citations.

From January 1, 2004, to June 4, 2024, a total of 2,843 publications related to nanoparticles in gynecologic cancers were identified in the WOSCC database based on the search criteria. Among these, 2,661 articles (93.6%) were research articles and 182 articles (6.4%) were reviews. As shown in **Figure 2**, the number of publications steadily increased from 2004 to 2020. Although there was a slight decrease after 2020, the overall trend over the past 20 years has been upward. Moreover, from 2021 to 2023, the annual number of publications remained around 290, indicating sustained attention to nanoparticles in the field of gynecologic cancers research. Furthermore, there has been a notable increase in citations, rising from 4 in 2004 to 13,090 in 2023. This increase in citations further underscores the significant scientific impact and relevance of findings from this research.

### 3.2 Analysis of countries/region and institutions

**Figure 3:**
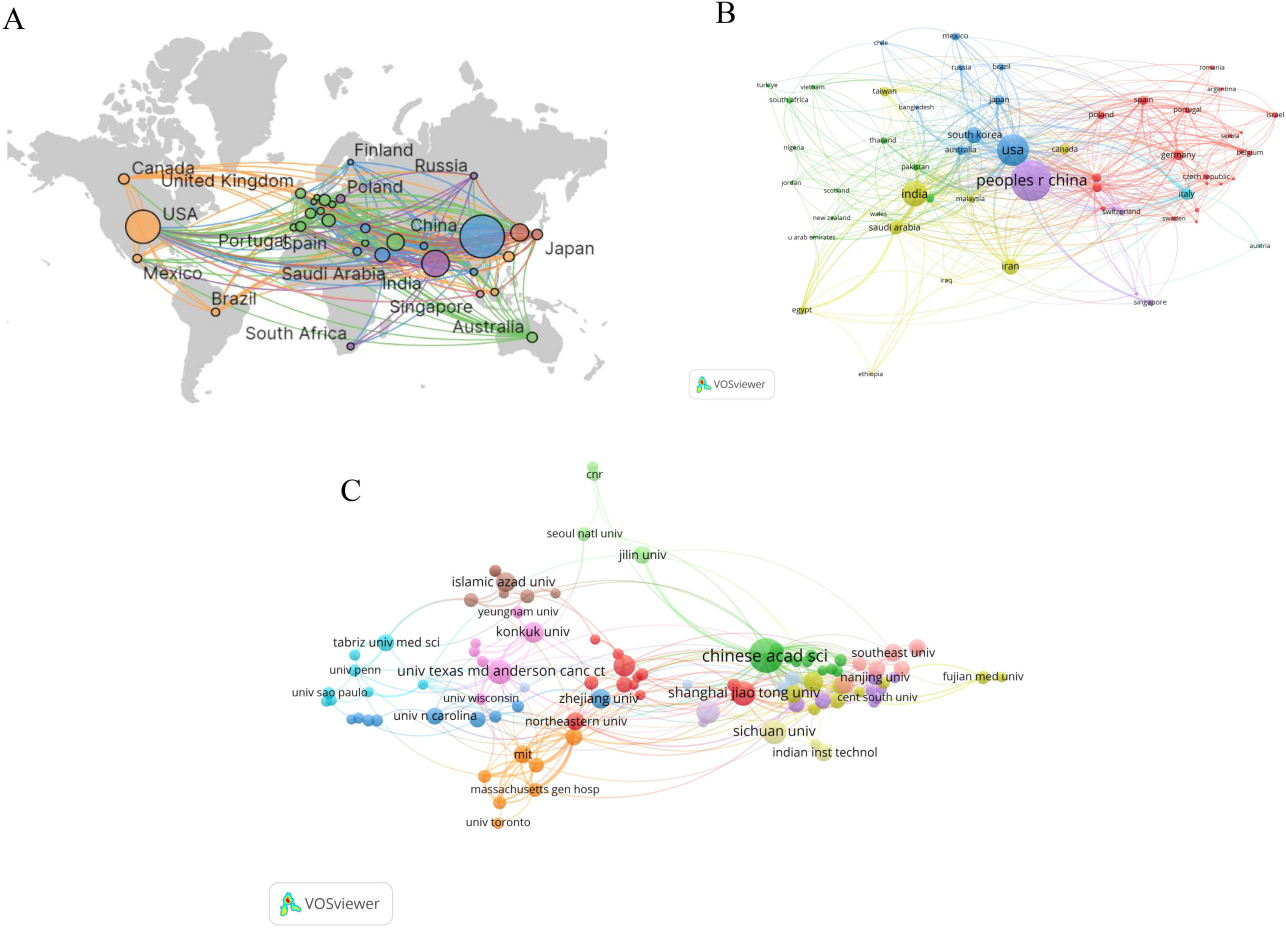
Visual map of countries/regions and institutions: **(A)** Geographic distribution map based on publication counts **(B)** Collaboration network visualization for countries/regions **(C)** Institutions with 10 or more publications.

Over the past 20 years, research on nanoparticles in gynecologic cancers has been conducted in 76 countries and regions. **Table 1** lists the top 10 countries/regions by publication count. China leads with 1,042 publications, followed by the United States (n = 624) and India (n = 399). **Figure 3A** depicts the global distribution of publications in this field, showing concentrations in North America, Europe, and Asia. The collaboration network visualization for countries/regions, as shown in **Figure 3B**, depicts total link strength (TLS), which reflects the intensity of collaboration or co-occurrence between nodes. The width of the connecting lines indicates the levels of international collaboration. According to **Table 1**, the top three countries ranked by TLS are the United States, China, and India. Centrality, which assesses the importance of nodes within a network, exceeds 0.1, indicating pivotal nodes with significant influence (28). Notably, despite ranking second in publication count, the United States exhibits the highest TLS and centrality, underscoring its predominant influence in this field (**Table 1**).

**Table 1:** Top 10 countries/regions and institutions in the field of nanoparticles in gynecologic cancers.

Rank	Country/Region				Institutions			
	Name	Count	TLS	Centrality	Name	Count	TLS	Centrality
1	China	1042	286	0.16	Chinese Academy of Sciences	105	102	0.17
2	USA	624	383	0.37	Shanghai Jiao Tong University	55	37	0.08
3	India	399	216	0.19	University of Texas MD Anderson Cancer Center	52	49	0.09
4	South Korea	168	137	0.05	Sichuan University	52	16	0.12
5	Iran	157	96	0.11	Fudan University	48	33	0.02
6	Saudi Arabia	113	164	0.12	King Saud University	42	13	0.13
7	Italy	87	81	0.06	Konkuk University	39	27	0.13
8	Germany	67	85	0.08	University of Chinese Academy of Sciences	35	50	0.02
9	Japan	61	74	0.02	Zhejiang University	35	9	0.03
10	Canada	61	41	0.01	Islamic Azad University	34	15	0.07

With regard to institutions, **Figure 3C** shows the institutions with 10 or more publications. The top publishing institutions include the Chinese Academy of Sciences (n=105), Shanghai Jiao Tong University (n=55), University of Texas MD Anderson Cancer Center (n=52) and Sichuan University (n=52) (**Table 1**). Of significance, the Chinese Academy of Sciences not only leads in publication quantity but also demonstrates the highest TLS and centrality, highlighting its central role in this research landscape.

### 3.3 Analysis of journals

Over the past 20 years, 637 journals have published 2,843 articles on nanoparticles in gynecologic cancers. Among these, 15 journals have published 30 or more articles. **Table 2** shows that the *International Journal of Nanomedicine* has the highest number of publications (n=97), followed by *ACS Applied Materials & Interfaces* (n=72) and *Journal of Materials Chemistry B* (n=53). The *International Journal of Nanomedicine* covers various aspects of nanotechnology applications in biomedicine, focusing on the potential clinical applications of nanoparticles in disease diagnosis, prevention, and treatment. In terms of impact factor, *ACS Nano* (n=32, IF=17.1) ranks highest among these journals. *ACS Nano*, as one of the top journals in the field of nanoscience, highlights research achievements in nanoscience and nanotechnology. The journal is interdisciplinary, spanning fields such as chemistry, physics, biology, and engineering.

**Table 2:** Top 15 journals in the field of nanoparticles in gynecologic cancers.

Rank	Journals		
	Name	Count	IF(2022)/JCR
**1**	*International Journal of Nanomedicine*	97	8.0/Q2
**2**	*ACS Applied Materials & Interfaces*	72	9.5/Q1
**3**	*Journal of Materials Chemistry B*	53	7.0/Q1
**4**	*Journal of Controlled Release*	46	10.8/Q1
**5**	*RSC advances*	42	3.9/Q2
**6**	*International Journal of Molecular Sciences*	38	5.6/Q1
**7**	*Journal of Biomedical Nanotechnology*	38	2.9/Q4
**8**	*Colloids and Surfaces B-Biointerfaces*	37	5.8/Q1
**9**	*International Journal of Pharmaceutics*	37	5.8/Q1
**10**	*Nanoscale*	37	6.7/Q1
**11**	*Biomaterials*	36	14.0/Q1
**12**	*Scientific Reports*	36	4.6/Q2
**13**	*ACS Nano*	32	17.1/Q1
**14**	*Nanomaterials*	31	5.3/Q2
**15**	*Molecular Pharmaceutics*	30	4.9/Q2

IF2022, Impact Factor 2022; JCR, Journal Citation Reports.

**Figure 4:**
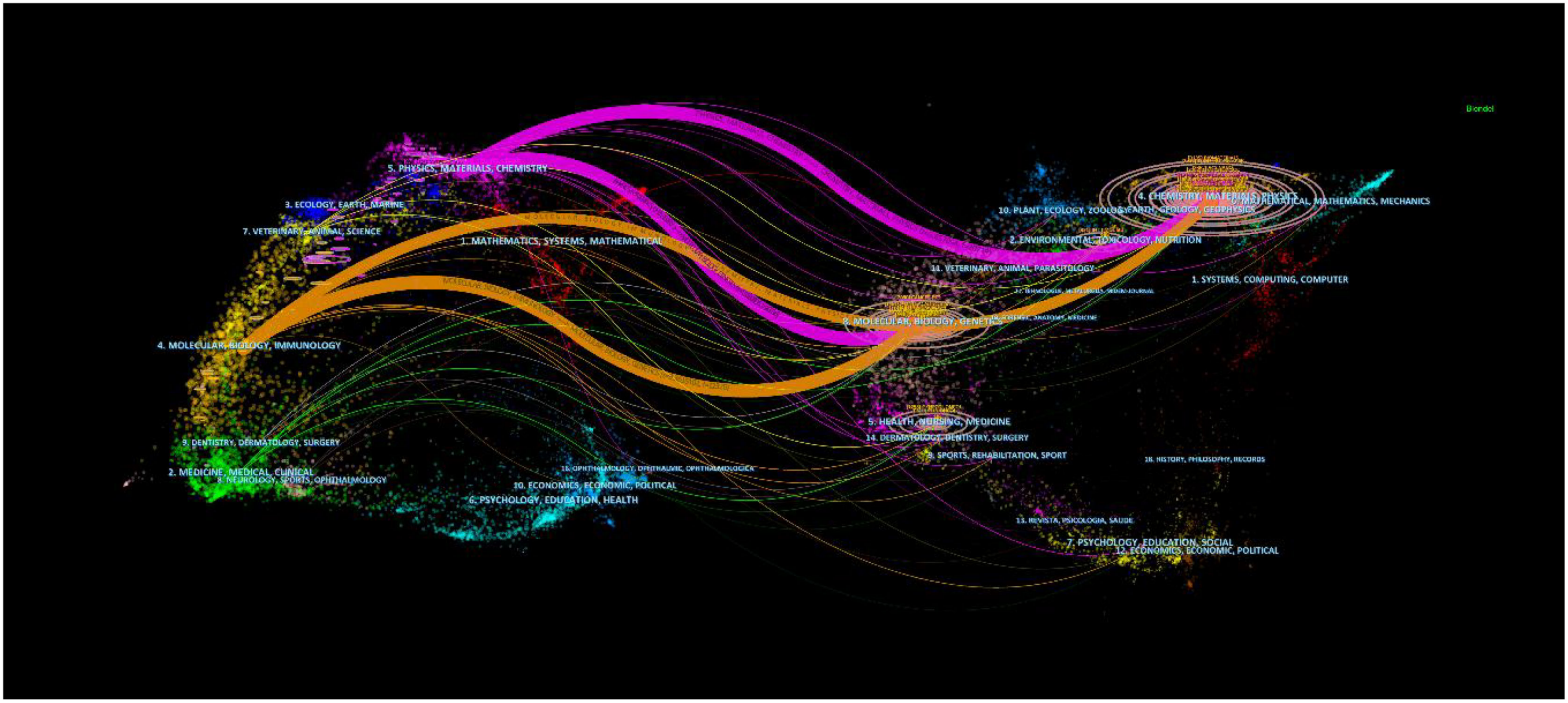
The dual-map overlay of journals in the field of nanoparticles in gynecologic cancers.

**Figure 4** presents a dual-map overlay visualization of academic journals, illustrating the relationships between journals. Citing journals are shown on the left side, and the cited journals are shown on the right side. Research published in journals focusing on chemistry/materials/physics and molecular/biology/genetics is frequently cited by journals focusing on physics/materials/chemistry and molecular/biology/immunology.

### 3.4 Analysis of authors and co-cited authors

**Figure 5:**
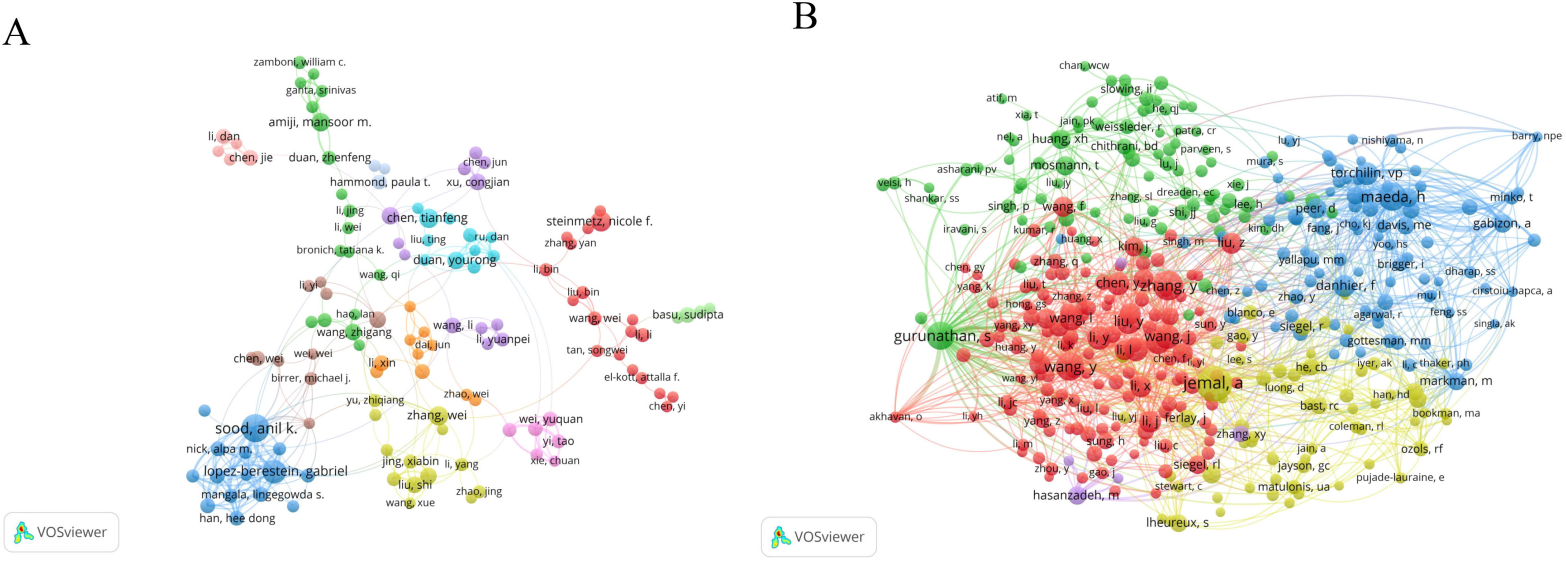
The network visualization map of authors: **(A)** Visualization analysis graph of author collaboration network; **(B)** The network visualization map of co-cited authors.

A total of 15,227 researchers have contributed articles in the field of nanoparticles in gynecologic cancers. Sood, A K has published the highest number of articles (n=30), followed by Lopez-Berestein G (n=22) and Steinmetz, NF (n=16). The collaboration network map among authors indicates close cooperation among them (**Figure 5A**). Co-cited authors are authors cited together in academic research, forming co-citation relationships (29). **Figure 5B** displays the network visualization map of co-cited authors. Siegel, RL ranks first in citation count (n=221), followed by Zhang, Y (n=213) and Gurunathan, S (n=191). **Table 3** outlines the citation counts for the top 10 co-cited authors, totaling over 1,700 citations, highlighting their significant influence in the field of nanoparticles in gynecologic cancers. It is noteworthy that Gurunathan, S is among the top ten authors in the current field and is also one of the top ten co-cited authors. In recent years, this author has made significant contributions to the biomedical application of nanoparticles such as graphene and silver nanoparticles (30–32). Furthermore, the author has delved deeply into the biological functions of exosomes, particularly their potential as delivery vehicles in cancer therapy and as emerging nanoplatforms in biomedical applications (33).

**Table 3:** Top 10 authors and co-cited authors in the field of nanoparticles in gynecologic cancers.

Rank	Authors			Co-cited authors		
	Name	Documents	TLS	Name	Citations	TLS
1	Sood, A K.	30	117	Siegel, Rl	221	2239
2	Lopez-Berestein G	22	98	Zhang, Y	213	2076
3	Steinmetz, NF	16	7	Gurunathan, S	191	2296
4	Zhang, W	14	17	Maeda, H	189	2252
5	Mei, l	13	23	Wang, Y	184	2065
6	Amiji, MM	13	20	Jemal, A	157	1511
7	Gurunathan, S	13	10	Wang, J	149	1675
8	Chen, T	13	4	Liu, Y	143	1637
9	Singh, M	13	0	Li, Y	137	1447
10	Duan, Y	12	29	Wang, X	133	1478

### 3.5 Analysis of co-cited references

**Table 4** outlines the top 10 co-cited references. These articles generally fall into three main themes: (1) cancer statistics, (2) nanoparticles in cancer therapy, and (3) research on cancer treatment methods and mechanisms. The two most co-cited articles are both from the prestigious journal “*CA: A Cancer Journal for Clinicians*,” focusing on global cancer statistics. The third most co-cited paper, published in *Nature Nanotechnology*, details a repository of nano-carriers and molecules for selective tumor targeting, emphasizing challenges in cancer treatment (34).

**Table 4:** Top 10 co-cited references in the field of nanoparticles in gynecologic cancers.

Rank	Co-cited reference	Journal	Years	Co-cited counts	IF(2022)/ JCR
1	Cancer Statistics, 2017	*CA: A Cancer Journal for Clinicians*	2017	138	254.7/Q1
2	Global cancer statistics	*CA: A Cancer Journal for Clinicians*	2011	107	254.7/Q1
3	Nanocarriers as an emerging platform for cancer therapy	*Nature Nanotechnology*	2007	91	38.3/Q1
4	Rapid colorimetric assay for cellular growth and survival: application to proliferation and cytotoxicity assays	*Journal of Immunological Methods*	1983	88	2.2/Q3
5	Tumor vascular permeability and the EPR effect in macromolecular therapeutics: a review	*Journal of Controlled Release*	2000	62	10.8/Q1
6	A new concept for macromolecular therapeutics in cancer chemotherapy: mechanism of tumoritropic accumulation of proteins and the antitumor agent smancs	*Cancer Research*	1986	62	11.2/Q1
7	Ovarian cancer statistics, 2018	*CA: A Cancer Journal for Clinicians*	2018	56	254.7/Q1
8	Nanoparticle therapeutics: an emerging treatment modality for cancer	*Nature Reviews Drug Discovery*	2008	49	120.1/Q1
9	Intraperitoneal cisplatin and paclitaxel in ovarian cancer	*New England Journal of Medicine*	2006	45	158.5/Q1
10	Determining the size and shape dependence of gold nanoparticle uptake into mammalian cells	*Nano Letters*	2006	44	10.8/Q1

**Figure 6:**
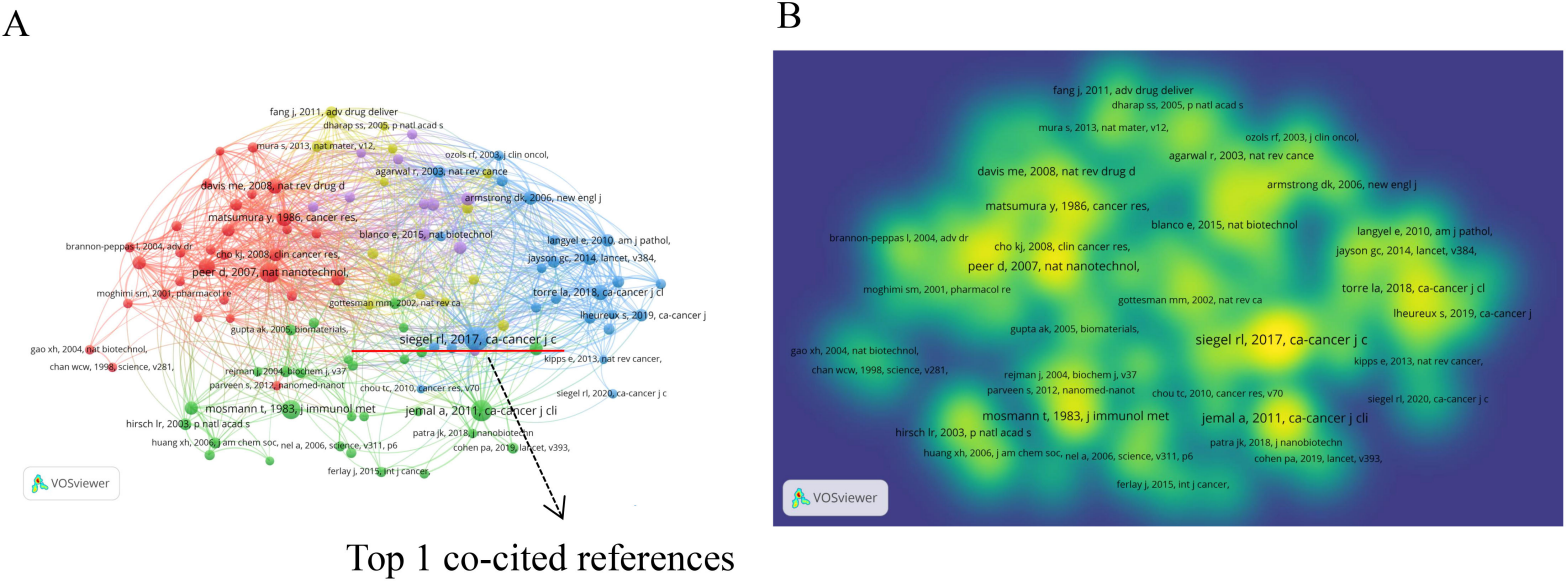
The network visualization map of references: **(A)** Co-citation network of references with 20 or more citations. **(B)** Density visualization of references co-cited 20 times or more.

**Figure 6A** displays the co-citation network of references with 20 or more citations, divided into 5 clusters. Cluster 1 (red) primarily discusses nanotechnology applications in cancer therapy. Cluster 2 (green) focuses on the mechanisms of nanoparticle action at the cellular level. Cluster 3 (blue) centers on tumor data statistics. Cluster 4 (yellow) emphasizes drug delivery systems. Cluster 5 (purple) addresses challenges in clinical applications of nanomedicine. **Figure 6B** depicts a density visualization of references co-cited 20 times or more, with colors closer to yellow indicating higher cited volumes. It is evident that cluster1 (red) and cluster3 (blue) contribute significantly to the cited volumes.

**Table 5** illustrates the top 25 references with the strongest citation bursts, with blue lines indicating the timeline and red segments representing burst intervals. Notably, 12 articles have experienced recent bursts, potentially indicating future trends in the field of nanoparticles in gynecologic cancers. Five articles focus on cancer data statistics, while four provide comprehensive reviews of two prominent types of gynecologic malignancies—ovarian and cervical cancers. The remaining three articles concentrate on cutting-edge therapeutic approaches for these diseases.

**Table 5:** Top 25 references with the strongest citation bursts.

References	Year	Strength	Begin	End	2004 - 2024
Gao XH, 2004, NAT BIOTECHNOL, V22, P969, DOI 10.1038/nbt994, DOI	2004	5.8	2007	2009	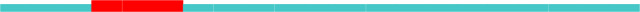
Jemal A, 2009, CA-CANCER J CLIN, V59, P225, DOI 10.3322/caac.21387, DOI	2009	12.01	2010	2012	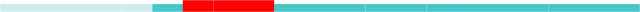
Davis ME, 2008, NAT REV DRUG DISCOV, V7, P771, DOI 10.1038/nrd2614, DOI	2008	6.12	2010	2013	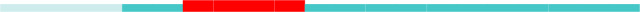
Fang J, 2011, ADV DRUG DELIVER REV, V63, P136, DOI 10.1016/j.addr.2010.04.009, DOI	2011	6.42	2013	2016	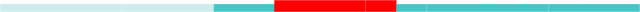
Siegel R, 2014, CA-CANCER J CLIN, V64, P9, DOI 10.3322/caac.21208, DOI	2014	7.81	2015	2019	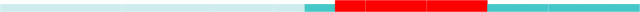
Mura S, 2013, NAT MATER, V12, P991, DOI 10.1038/NMAT3776, DOI	2013	7.51	2015	2018	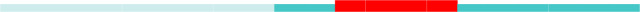
Blanco E, 2015, NAT BIOTECHNOL, V33, P941, DOI 10.1038/nbt.3330, DOI	2015	8.07	2016	2020	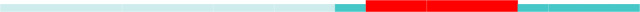
Bertrand N, 2014, ADV DRUG DELIVER REV, V66, P2, DOI 10.1016/j.addr.2013.11.009, DOI	2014	8.06	2016	2019	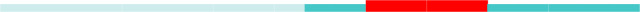
Ferlay J, 2015, INT J CANCER, V136, PE359, DOI 10.1002/ijc.29210, DOI	2015	7.33	2016	2020	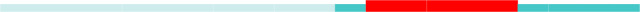
Wilhelm S, 2016, NAT REV MATER, V1, P0, DOI 10.1038/natrevmats.2016.14, DOI	2016	9.66	2017	2021	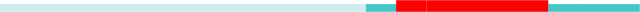
Shi JJ, 2017, NAT REV CANCER, V17, P20, DOI 10.1038/nrc.2016.108, DOI	2017	6.08	2018	2019	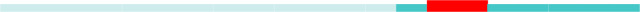
Torre LA, 2018, CA-CANCER J CLIN, V68, P284, DOI 10.3322/caac.21456, DOI	2018	10.94	2019	2024	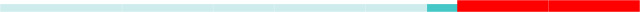
Reid BM, 2017, CANCER BIOL MED, V14, P9, DOI 10.20892/j.issn.2095-3941.2016.0084, DOI	2017	7.48	2019	2022	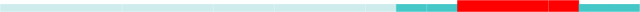
Bowtell DD, 2015, NAT REV CANCER, V15, P668, DOI 10.1038/nrc4019, DOI	2015	5.89	2019	2020	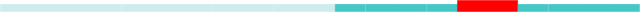
Cohen PA, 2019, LANCET, V393, P169, DOI 10.1016/S0140-6736(18)32470-X, DOI	2019	8.08	2020	2024	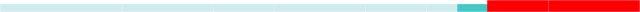
Stewart C, 2019, SEMIN ONCOL NURS, V35, P151, DOI 10.1016/j.soncn.2019.02.001, DOI	2019	8.89	2021	2024	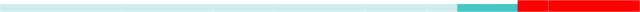
Lheureux S, 2019, LANCET, V393, P1240, DOI 10.1016/S0140-6736(18)32552-2, DOI	2019	7.85	2021	2024	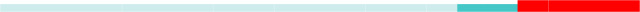
Patra JK, 2018, J NANOBIOTECHNOL, V16, P0, DOI 10.1186/s12951-018-0392-8, DOI	2018	7.55	2021	2024	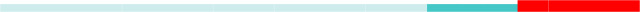
Arbyn M, 2020, LANCET GLOB HEALTH, V8, PE191, DOI 10.1016/S2214-109X(19)30482-6, DOI	2020	7.17	2021	2024	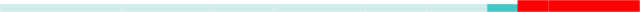
Barani M, 2021, LIFE SCI, V266, P0, DOI 10.1016/j.lfs.2020.118914, DOI	2021	6.13	2021	2024	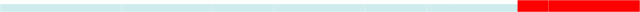
Pokhriyal Ruchika, 2019, BIOMARK CANCER, V11, P1179299X19860815, DOI 10.1177/1179299X19860815, DOI	2019	6.03	2021	2024	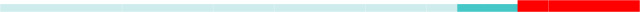
Sung H, 2021, CA-CANCER J CLIN, V71, P209, DOI 10.3322/caac.21660, DOI	2021	24.7	2022	2024	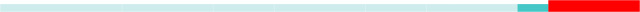
Lheureux S, 2019, CA-CANCER J CLIN, V69, P280, DOI 10.3322/caac.21559, DOI	2019	12.1	2022	2024	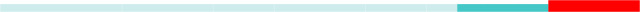
Siegel RL, 2022, CA-CANCER J CLIN, V72, P7, DOI 10.3322/caac.21708, DOI	2022	7.21	2022	2024	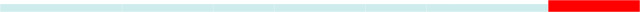
Kuroki L, 2020, BMJ-BRIT MED J, V371, P0, DOI 10.1136/bmj.m3773, DOI	2020	7.11	2022	2024	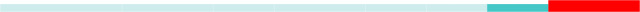

### 3.6 Analysis of keywords

**Figure 7:**
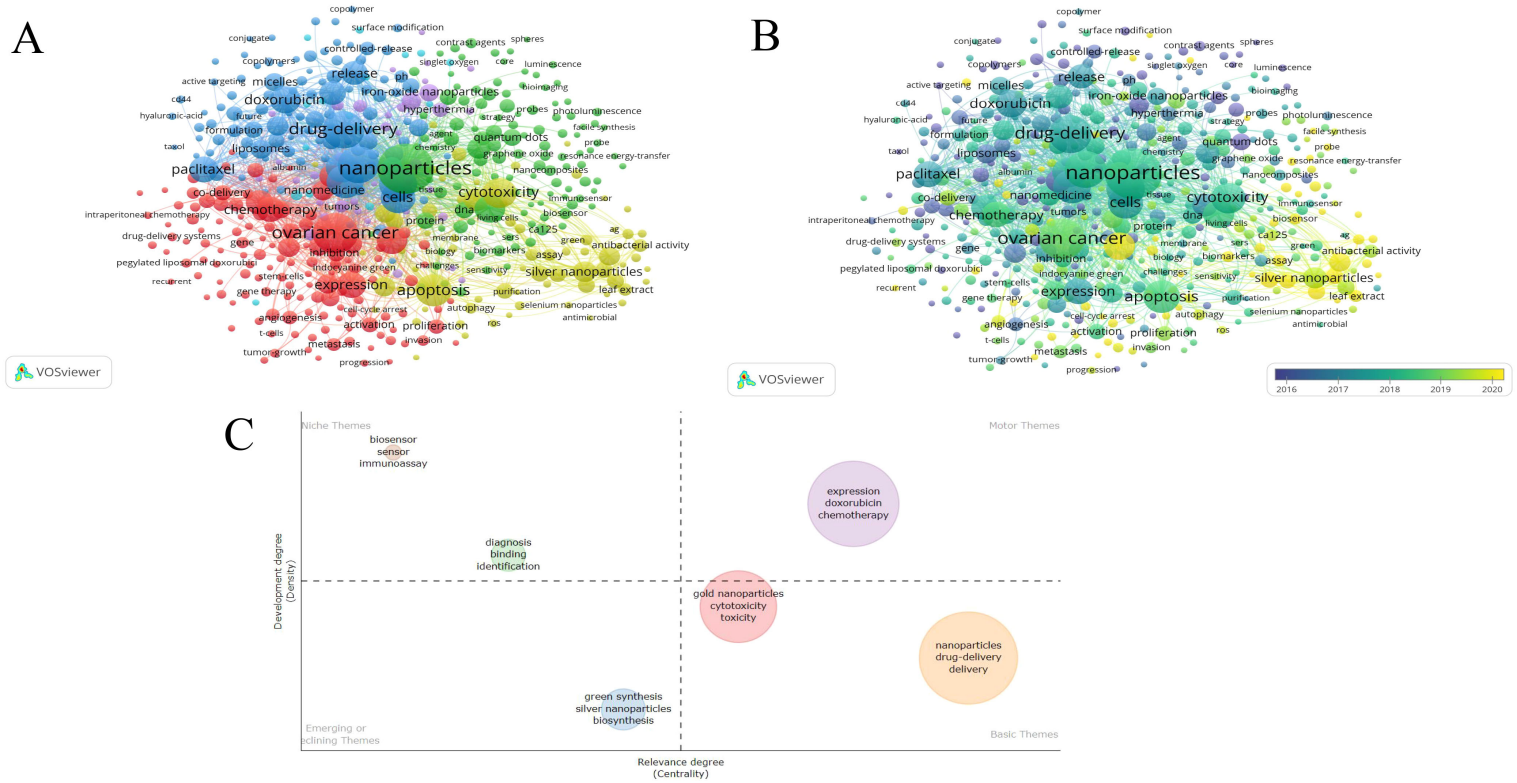
The network visualization map of keywords in the field of nanoparticles in gynecologic cancers: **(A)** Network visualization of keywords appearing 10 times or more. **(B)** Overlay visualization of keywords appearing 10 times or more. **(C)** Development trend and importance of hot spots.

In the field of nanoparticles research related to gynecologic malignancies, **Table 6** present the frequency distribution of the most commonly occurring 10 keywords. Among the top 10 keywords, excluding those directly related to the search terms, current popular topics include drug delivery, cells, therapy, apoptosis, expression, and release. **Figure 7A** presents the keyword co-occurrence visualization map with keywords appearing 10 times or more, comprising 484 nodes and 25,920 links. The size of each node representing a keyword is directly proportional to its frequency of occurrence. The graph can be divided into four major clusters based on different colors. The red cluster represents common gynecologic malignancies and treatment modalities, the green cluster includes commonly used nanoparticles in gynecologic malignancies, the blue cluster focuses on drug delivery systems, and the yellow cluster primarily discusses clinical mechanisms of nanoparticle action at the cellular level. **Figure 7B** displays an overlay visualization where keywords closer to yellow indicate recent significant impact, suggesting heightened attention on “silver nanoparticles”, “green synthesis” and “antibacterial” in recent years.

**Table 6:** Top 10 keywords in the field of nanoparticles in gynecologic malignancies.

Rank	Keyword	Occurrences
1	nanoparticles	518
2	drug-delivery	396
3	delivery	382
4	*in-vitro*	319
5	cells	278
6	therapy	247
7	cancer	246
8	apoptosis	215
9	expression	190
10	release	167

In addition to current and past keywords, future potential hotspots and development trends are analyzed in **Figure 7C**. The first quadrant signifies crucial and well-developed issues, the second quadrant indicates well-developed but currently less significant topics, the third quadrant represents emerging or declining topics, and the fourth quadrant denotes important but less developed issues in the field. The first quadrant includes the keywords expression, doxorubicin, and chemotherapy, which signify advancements in nanotherapy offering new avenues for refining traditional chemotherapy methods. In the fourth quadrant, keywords such as gold nanoparticles, cytotoxicity, toxicity, nanoparticles, drug-delivery, and delivery underscore the application of nanoparticles in drug delivery, while emphasizing the critical need for further exploration in related cytotoxicity and toxicity assessment studies.

**Table 7** illustrates the top 25 keywords with the strongest citation bursts. Keywords such as “antioxidant,” “cervical cancer,” “endometrial cancer,” “antibacterial,” “epithelial ovarian cancer,” “green synthesis,” “gold nanoparticles,” and “cisplatin resistance” have garnered significant attention in recent years and are likely to continue to attract interest. This highlights ongoing interests and signifies potential trends in the field of nanoparticles in gynecologic malignancies.

**Table 7:** Top 25 keywords with the strongest citation bursts.

Keywords	Year	Strength	Begin	End	2004 - 2024
*in vivo*	2005	8.66	2005	2015	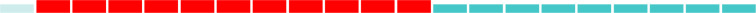
quantum dots	2005	6.62	2005	2013	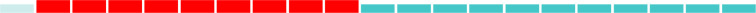
cancer	2005	6.39	2006	2010	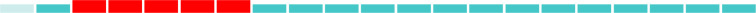
carrier	2006	5.61	2006	2012	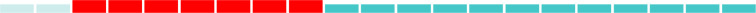
anticancer drug	2007	6.91	2007	2016	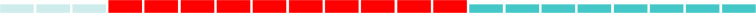
nanocrystal	2008	5.68	2008	2012	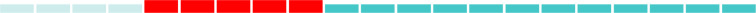
particle	2009	11.83	2009	2014	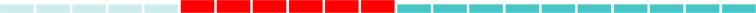
magnetic nanoparticle	2009	9.36	2009	2016	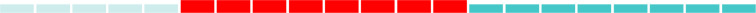
receptor	2009	6.94	2009	2014	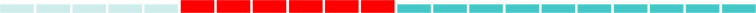
angiogenesis	2010	7.89	2010	2013	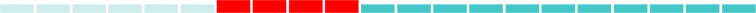
toxicity	2008	6.07	2011	2014	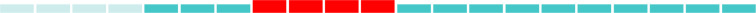
contrast agent	2012	6.21	2012	2016	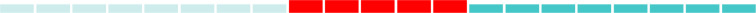
micelle	2012	5.89	2015	2016	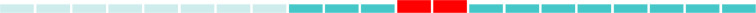
ph	2012	5.32	2016	2018	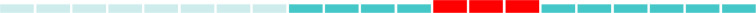
tumor targeting	2017	5.31	2017	2020	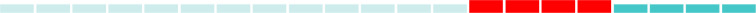
antioxidant	2016	8.92	2019	2024	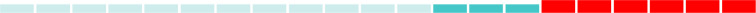
peptide	2009	6.46	2019	2020	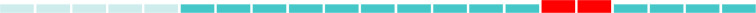
cervical cancer	2005	17.75	2021	2024	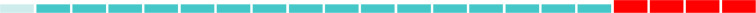
silver nanoparticle	2014	8.81	2021	2022	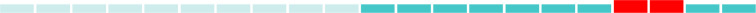
endometrial cancer	2021	5.72	2021	2024	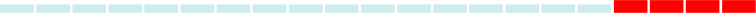
antibacterial	2014	6.07	2022	2024	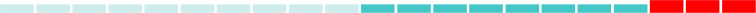
epithelial ovarian cancer	2022	6.04	2022	2024	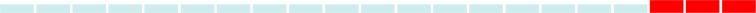
green synthesis	2014	5.92	2022	2024	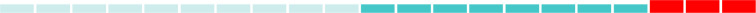
gold nanoparticles	2005	5.7	2022	2024	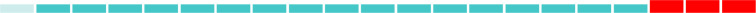
cisplatin resistance	2020	5.47	2022	2024	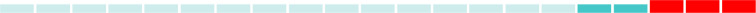

## 4 Discussion

### 4.1 General information

Publications are crucial indicators for assessing the academic vitality and developmental trends of research fields, providing an objective reflection of research dynamics in specific domains (35). **Figure 2** shows there has been a significant increase in publications related to nanoparticles in gynecologic malignancies over the past two decades. Approximately 85.72% (n=2437) of these publications are concentrated in the last decade. Notably, while the United States ranks second in publication volume, accounting for only about 60% of China’s output, its centrality is more than twice that of China’s, indicating substantial influence in the field. This phenomenon can be attributed to factors such as the high burden of gynecologic malignancies in the United States and robust healthcare investments (36, 37). The United States is a global leader in the field of nanomedicine, accounting for 46% of the global market share in 2016 (38). In 2018, the National Institutes of Health (NIH) invested an estimated $445 million in nanomedicine research (39). This substantial financial investment has provided a solid foundation for the development of nanomedicines, particularly in the area of anticancer therapies. While nanotherapy may offer an efficient treatment option, the high research and production costs may restrict its widespread use in resource-limited countries. Developing countries could reduce production and supply costs for nanotherapy through supportive policies, such as tax incentives and patent protection adjustments. Furthermore, establishing international collaboration platforms to share key technologies and research outcomes can help lower research costs.

What’s more, the Chinese Academy of Sciences not only leads in terms of publication volume but also stands out as the institution with the greatest influence. Although it collaborates with institutions such as the Massachusetts Institute of Technology, University of California, Los Angeles, and Dana-Farber Cancer Institute, most collaborations remain within China, including Shanghai Jiao Tong University, Sichuan University, Fudan University, and Zhejiang University. Considering the serious health threat posed by gynecologic malignancies and the tremendous potential of nanoparticles in treating these cancers, strengthening international collaborations among institutions is imperative to advance research in this field.

A total of 637 academic journals have published research articles on nanoparticles in gynecologic malignancies, with the top 15 journals accounting for 23.29% of the publication output. The *International Journal of Nanomedicine* leads in publication volume; however, there remains a shortage of high-impact factor journals in this field. Sood, A K and Siegel, Rl are respectively the most prolific and most cited authors in this domain. Sood, A K’s research includes liposomal nanoparticles for delivering small interfering RNAs(siRNA) in cancer therapy and the application of RNA-targeted therapy in ovarian cancer treatment. Liposomal nanoparticles enhance stability and bioavailability, effectively delivering siRNA to tumor tissues for RNA-targeted therapy, which plays a crucial role in treating gynecologic malignancies (40, 41). And Siegel RL has made outstanding contributions to cancer statistics (42).

**Table 5** lists the top 25 references with the strongest citation bursts. The three articles with the highest burst strengths are all from the prestigious journal *CA: A Cancer Journal for Clinicians*. The strongest burst is attributed to the publication by Sung H et al. on the 2020 global cancer statistics (43), followed by the research on the evolving management of epithelial ovarian cancer by Lheureux S et al. (44), and the relevant study on cancer data statistics from 2009 by Jemal A et al (45). Excluding literature solely focused on the current status of gynecologic tumors and considering both the strength and time of burst, two articles deserve particular attention. The first article, authored by Patra JK et al., comprehensively reviews the discovery and application of nanoparticles, emphasizing their crucial roles in cancer therapy and diagnosis. For instance, oleic acid-coated iron oxide nanoparticles can be employed for cancer diagnosis using near-infrared, while hyaluronic acid-coated iron oxide can be used for cancer treatment (46). The second article, authored by Barani M et al., focuses on nano-scale drug delivery systems in ovarian cancer treatment, providing robust scientific evidence for targeted therapy in ovarian cancer. The utilization of nanobiosensors to evaluate biomarkers has illuminated a novel path for guiding treatment in ovarian cancer patients (47).

### 4.2 Research hotspots

Keywords condense the essence and core of a paper, representing the central themes and topics of research. Analyzing keyword frequency and burst detection can reflect research hotspots and future trends in a field. Based on keyword clustering, we elaborated on the following two main aspects in detail:

(1) Nanoparticles for drug delivery

Targeted drug delivery systems generally refer to the use of nanomedicine carriers, such as nanoparticles, administered via routes like vascular injection, to deliver drugs to specific sites of disease (48). Nanocarriers demonstrate potential in delivering various anticancer drugs and enhancing treatment efficacy (49), achieving active targeting from passive delivery, prolonging drug circulation in the body, and effectively eliminating cancer cells (50). Currently, common nanocarriers used for drug delivery in gynecologic malignancies include liposome nanoparticles, polymer nanoparticles, and inorganic nanoparticles. Ledezma-Gallegos et al. developed liposomal nanoparticles co-encapsulating cisplatin and mifepristone, demonstrating significant cytotoxic effects against cervical cancer cells, thereby enhancing chemotherapy efficacy (51). Wang et al. also found that liposomes loaded with miRNA-1284 and cisplatin enhance the treatment of cervical cancer (52). Li et al. improved the tumor microenvironment and enhanced the efficacy of drugs against gynecologic malignancies using cancer cell membrane-modified nanoparticles loaded with dexamethasone (53). Although research confirms that liposome nanoparticles can enhance the treatment of gynecologic cancers, either directly or indirectly, their effectiveness in encapsulating highly hydrophobic drugs, such as paclitaxel commonly used in ovarian cancer treatment, is limited. This limitation arises because the liposomal structure resembles that of biofilms, which complicates drug retention within the liposomes (54–56). Furthermore, Clinical translation still faces numerous challenges, such as low drug-loading capacity and high uptake rates in the liver and spleen, which may affect treatment safety (56, 57). Polymer nanoparticles exhibit characteristics such as water solubility, excellent stability, and biocompatibility. Researchers like Wang demonstrated the significance of folate-modified, pH-sensitive polymer nanoparticles co-delivering carboplatin and paclitaxel in both *in vitro* and *in vivo* models for treating cervical cancer (58). However, several unresolved challenges remain. First, the synthesis of polymer nanoparticles is complex and costly, potentially hindering large-scale clinical applications. Moreover, further investigation is required to ensure that polymer nanoparticles remain stable *in vivo*, evade immune recognition, and minimize potential toxicity (59, 60). Inorganic nanoparticles mainly include gold, silver, oxides, etc. Due to their magnetic, optoelectronic, antimicrobial, and plasmonic properties, they offer unique advantages in diagnostics, lymph node imaging, fluorescence tracking, etc (61, 62). For instance, silver nanoparticle-modified amine-functionalized mesoporous silica nanoparticles show significant potential in delivering doxorubicin for cervical cancer treatment (49). However, clinical application is limited due to inherent toxicity of some inorganic nanoparticles (63). Given the distinct advantages and drawbacks of different types of drug-delivery nanocarriers, the number of nano-drugs applied clinically for treating gynecologic malignancies remains limited. For example, Food and Drug Administration (FDA) approval of Doxil (liposomal doxorubicin) widely used for treating metastatic ovarian cancer represents a major breakthrough in the field. In summary, nanomedicine carriers for treating gynecologic cancers, including liposomes, polymers, and inorganic nanoparticles, hold immense potential and face significant challenges in enhancing drug delivery efficiency and treatment efficacy. Currently, their clinical application remains limited. Enhanced interdisciplinary collaboration and the accumulation of clinical data will help to more comprehensively evaluate the actual effectiveness of nanodrug carriers in treating gynecologic cancers.

(2) Nanoparticles in the research of gynecologic cancers

Through keyword clustering analysis, we found that magnetic, iron-oxide, silver, and gold nanoparticles are frequently mentioned in the context of gynecological cancers. Taking ovarian cancer, which has the highest mortality and poorest prognosis among gynecological malignancies, as an example, early-stage detection is challenging due to the absence of specific clinical symptoms and sensitive biomarkers. Traditional tissue biopsy is considered the gold standard for diagnosis, but it can lead to cancer cell dissemination into the peritoneum during early ovarian cancer tissue sampling (64, 65). Nanoparticles demonstrate promising performance in these aspects. For instance, Pan et al. developed a simple and cost-effective method using folate-modified fluorescent magnetic nanoparticles to capture and identify circulating tumor cells in ovarian cancer patients, aiding in metastasis diagnosis (66). Similarly, Liu et al. utilized folic acid conjugated magnetic iron oxide nanoparticles to bind to overexpressed folate receptor and thus stably attach to the surface of ovarian cancer cells, rendering the cells magnetic and thus enabling the possibility of early detection of metastatic ovarian cancer cells (67). In addition to the aforementioned nanoparticles, silver and gold nanoparticles also play crucial roles. These nanoparticles not only serve as popular keywords but also represent future research trends and hotspots (see **Figure 7C**, **Table 7**). *In vitro* experiments demonstrate that triangular silver nanoparticles significantly inhibit ovarian cancer cell proliferation and growth, suggesting their potential as a future therapeutic approach (68). Silver nanoparticles exert cytotoxic effects by activating the p53 gene, thereby suppressing cancer cell growth. Interactions between gemcitabine and silver nanoparticles exhibit cytotoxicity in ovarian cancer cells (69, 70). Curcumin-coated silver nanoparticles act as sensitizers for cisplatin chemotherapy, potentially reducing side effects and enhancing efficacy (70, 71). Gold nanoparticles, similar to silver nanoparticles, enhance sensitivity of ovarian cancer cells to cisplatin (72). Upon binding with cisplatin, gold nanoparticles not only augment cytotoxic effects against tumor cells but also mitigate toxicity towards normal cells (73). Apart from enhancing cisplatin’s functionality, gold nanoparticles arrest cancer cells in the radiosensitive cell cycle phase (G2/M), further sensitizing them to radiation therapy (74, 75).

Beyond ovarian cancer, superparamagnetic iron oxide nanoparticles demonstrate extensive potential in the treatment of other gynecological malignancies such as cervical, endometrial, and vulvar cancers. This novel magnetic resonance lymphography technique utilizes iron oxide nanoparticles, avoiding the use of radioactive isotopes, and enables detection by magnetic resonance imaging devices following lymphatic transport to lymph nodes. However, further clinical application and research are needed to fully develop this technology (76–78). Additionally, silver nanoparticles enhance camptothecin’s therapeutic efficacy against cervical cancer cells compared to monotherapy (79). L-histidine-terminated silver nanoparticles also show promise as a potential effective treatment for cervical cancer (80). Furthermore, silver nanoparticles bind with DNA for detection purposes in gynecological malignancies (81). Gold nanoparticles not only directly induce BAX gene expression to induce apoptosis in cervical cancer cells but also form complexes with antibodies as Au@SiO2 nanoparticles for potential therapeutic applications in cervical cancer (17, 82). Moreover, gold nanoparticles act as sensitizers in radiotherapy and ultrasound irradiation, exhibiting synergistic effects (83). Therefore, nanoparticles such as iron oxide, silver, and gold nanoparticles demonstrate multifaceted clinical applications in gynecological malignancies.

Overall, silver and gold nanoparticles show diverse clinical potential in the treatment of gynecologic malignancies. However, most studies remain confined to laboratory and *in vitro* research, with insufficient clinical trial data to fully support their safety and efficacy. Challenges to broader application include their *in vivo* stability, potential toxicity, and possible immune responses (84). Balancing their anticancer effects with potential side effects to facilitate safer and earlier clinical use remains a pressing issue.

### 4.3 Future trends

Based on emerging keyword trends (see **Table 7**) and a hotspot analysis (**Figure 7C**), this study outlines three future trends in nanoparticles for gynecologic cancers as follows:

(1) Silver and gold nanoparticles remain pivotal in future research on nanoparticles in gynecologic cancers. These nanoparticles not only serve as sensitizers for radiotherapy and chemotherapy but also, due to their unique biological properties, exhibit effectiveness in improving wound healing processes post-surgery, with activities against bacteria, fungi, viruses, and parasites (85, 86). However, silver and gold nanoparticles may also exert toxicity on normal cells, particularly hepatocytes. Therefore, further research on these nanoparticles is essential.
(2) Transitioning nanoparticles from preclinical stages to clinical application remains a crucial challenge. Although silver and gold nanoparticles have shown promising results in both animal and cell experiments (87, 88), they have not yet been approved by the U.S. Food and Drug Administration for the treatment of gynecologic malignancies. Currently, the types of nanoparticles used in clinical trials are limited, with most studies focusing on approved products. Research on nanoparticles for clinical trials remains insufficient and requires further advancement (89). Future researches need further bridge the gap between the theoretical potential and practical clinical application of nanoparticles to facilitate their viability as treatments for gynecologic cancer.
(3) Green synthesis represents a crucial approach for synthesizing nanoparticles in the future. It is a method that uses natural resources and eco-friendly approaches to produce materials, aiming to minimize the impact of toxic chemicals. Compared to traditional methods, green synthesis utilizes the reducing potential of compounds found in biological organisms, offering advantages such as environmental friendliness, sustainability, and minimal toxicity of by-products (90). However, current green synthesis methods often operate under mild conditions and require further optimization regarding pH, temperature, and other chemical parameters (91). Moreover, green synthesis of nanomedicines still faces challenges in terms of reproducibility, relatively high costs, product standardization, and scalability (92). Therefore, further research is needed to optimize cost-effective methods for the green synthesis of nanomaterials.

### 4.4 Limitation

Several limitations exist in this study. Firstly, we exclusively relied on the WoSCC database for our search. While WoSCC is commonly used for bibliometric analysis, it may not encompass relevant literature from other databases, which could lead to the omission of some pertinent studies. Secondly, our study only considered English-language articles, potentially overlooking non-English publications and thereby potentially reducing credibility. Lastly, articles included in our study spanned from January 1, 2004, to June 4, 2024, and the WoSCC database is continuously updated, suggesting that recently published literature may be underestimated.

## 5 Conclusion

This study provides a comprehensive and objective bibliometric analysis of the application of nanoparticles in the field of gynecologic malignancies. The results indicate that the application of nanoparticles in this area has broad prospects and is developing rapidly. In terms of the number of publications, China and the Chinese Academy of Sciences are leading, while the United States dominates in academic influence. Future research, whether at the individual, institutional, or national level, should strengthen international exchange and collaboration to promote scientific progress and technological innovation in this field. Meanwhile, it is found that silver nanoparticles and gold nanoparticles show great potential in therapy, while green synthesis has received high attention as a sustainable preparation method for nanoparticles. Research hotspots focus on the drug delivery and diagnostic applications of nanoparticles, with future trends pointing toward the optimization of synthesis technologies and the translation of preclinical research into clinical applications. Strengthening international cooperation and standardizing technical protocols will help accelerate the clinical application of nanotechnology in the treatment of gynecologic cancers.

## Data Availability

The original contributions presented in the study are included in the article. Further inquiries can be directed to the corresponding authors.
